# Comparing categorical variables in clinical and experimental studies

**DOI:** 10.1590/1677-5449.20210225

**Published:** 2022-04-01

**Authors:** Anna Carolina Miola, Hélio Amante Miot

**Affiliations:** 1 Universidade Estadual Paulista “Júlio de Mesquita Filho” – UNESP, Faculdade de Ciências Médicas e Biológicas de Botucatu, Botucatu, SP, Brasil.

Many studies of a quantitative nature use qualitative variables, within both the biomedical sciences and the social sciences. These variables are also known as categorical and their magnitude is expressed in terms of the frequency with which each of their categories occurs. Qualitative variables can be subdivided into dichotomous (for example, sex, death, cure), ordinal (for example, cancer staging, pulse amplitude, functional class, phototype, anesthetic risk) or polytomous/multinomial (for example, sexual orientation, ABO type, marital status, religion, race, aneurysm type, type of chronic ulcer).^1^-^3^

When qualitative variables are employed, the phenomenon measured can be represented as the percentage of occurrence in each category, and subgroups should be compared in terms of the proportion of the sample that is attributed to each class.^3^ There is an extensive literature on techniques for statistical analysis of qualitative variables;^4^-^6^ whereas this text will deal with comparison of proportions between categorical variables. Comparative analysis of proportions between subgroups employs different concepts from parametric statistics, providing lower statistical power (larger type II error) in analogous situations, such as when a quantitative variable (for example, age) is categorized (for example, < 30 years, 30–59 years, ≥ 60 years).^7^,^8^

According to frequentist statistics,^9^ the probability of a proportion of events selected at random, without replacement of cases, can be generalized from the chi-square distribution, while Pearson’s chi-square test is based on the difference between the frequencies observed and the frequencies ideally expected for each category and can be used to compare how well a sample fits a known distribution (for example, for comparison with the literature) or independence between different samples.^10^ Despite the popularity of Pearson’s chi-square test, other methods such as the G test (likelihood ratio) and the Goodman test (contrasts between proportions) are also used to compare proportions. However, absolute superiority between them has not yet been systematically defined.^11^-^14^

An observed proportion’s fit can be compared to a description from the literature or a theoretical prediction (for example, expression of a phenotype according to segregation of a gene).^15^ For example, Tamega et al.^16^ studied ABO and Rh blood typing of 69 patients with lupus erythematosus, comparing them against the expected frequencies of these categories among blood donors at the institution. Pearson’s chi-square test (of fit) returned a *p*-value 0.081 for ABO types and a *p*-value of 0.721 for Rh types, accepting the hypothesis that these blood type classes did not differ from what was expected in the local population.^16^

In clinical-epidemiological research, it is highly usual to present an initial descriptive table containing demographic data on subgroups, in order to demonstrate their homogeneity. For example, Amiri et al.^17^ included 110 cases and 110 controls in a cross-sectional study to test associations between anthropometric indices and type 2 diabetes mellitus. Of these diabetic patients, 75 (51%) were female, whereas there were only 72 women (49%) in the control group. In this sample, the difference in proportion between the groups (2%) was not considered significant (*p*-value = 0.668) for this dichotomous variable according to Pearson’s chi-square test (of independence).

While versatile, Pearson’s chi-square test has inadequate performance (larger type I error) in smaller samples (*n* ≤ 40), especially in which > 20% of expected values are ≤ 5, which is relatively common in biomedical research scenarios. Several procedures are recommended in this situation, ranging from combining categories to increase the predicted value (for example, dichotomizing skin colors as white vs. not white, combining less common B blood groups with AB) or using other statistical tests.

There is an intense academic debate about which analytical strategies should be adopted for situations in which Pearson’s chi-square test is contraindicated, while different tests for categorical variables can behave differently depending on the manner in which the data are collected (randomized or not), since a large proportion of studies do not employ a completely randomized sampling structure.^18^-^20^ The Barnard and Boschloo exact tests are two examples that correct for these limitations for 2 × 2 contingency tables.^21^,^22^ In turn, the G test (with Williams’ correction) can be used for multinomial comparisons in situations in which Pearson’s chi-square test is contraindicated.^21^,^23^ Estimates of the (exact) *p*-value using resampling (bootstrapping) or Monte Carlo simulation are also effective in cases with modest samples or subgroups with a low predicted rate of occurrence.^19^,^24^

Fisher’s exact test is cited in many texts as a solution for cases in which Pearson’s chi-square test is not indicated, but it inflates the type II error, in addition to being based on a conditional probability model, which contrasts with what is usually proposed in biomedical research biomedical research (variable marginal totals).^25^,^26^ Along the same lines, correcting Pearson’s chi-square test with Yates’ procedure is excessively conservative in 2 × 2 tables. Use and interpretation of these tests should be parsimonious when they return *p*-values close to the significance level.^22^,^24^

For more complex designs, involving interaction between more than two categorical variables or multivariate adjustments in which the dependent variable is categorical, other methods of analysis can be used, such as Poisson regression (log-linear), logistic regression, and multinominal regression, which, as with Pearson’s chi-square test, are penalized in cases with low frequencies in subgroups. On the other hand, multivariate methods, such as multiple correspondence analysis, are unaffected by the contingencies of tests of hypotheses and can support exploratory analyses of categorical data.^4^,^27^,^28^ Meanwhile, the problems linked to analysis of ordinal data and calculation of sample sizes for studies involving proportions have been covered previously.^2^,^29^-^32^

When the results of comparisons of multinomial variables are significant, it remains to be determined which of the internal proportions diverge from the expected, since the test result (for Pearson’s chi-square test, for example) refers to the overall behavior of the proportions, so it is necessary to proceed to a *post hoc* analysis of the subcategories. Analysis of the residuals of the contingency table (standardized and adjusted) is a widely-used strategy that returns a *Z* statistic (Zres) for each proportion found, enabling multiple comparison between them to identify which specific variables most contribute to the result observed in the global test.^33^ By analyzing the residuals shown in Table 1, it can be concluded that cancer patients referred from clinics exhibited more incidental tomographic diagnoses of pulmonary thromboembolism than those admitted via the emergency room, however, no differences were found in the proportions from inpatients and those form ICU.^34^

**Table 1:** Analysis of residuals in data from Carneiro et al.^34^ on the origin of cancer patients with pulmonary thromboembolism (PTE) on computed tomography of the thorax, when the finding was incidental or there was a prior suspicion.

**Origin**	**No suspicion of PTE**		**PTE suspected previously**	
(*n* = 48)	Zres (*p*-value)	(*n* = 60)	Zres (*p*-value)
Clinic	28 (59%)	*+5.1 (<0.001)*	7 (12%)	*-5.1 (<0.001)*
Wards	16 (33%)	-0.2 (0.856)	21 (35%)	+0.2 (0.856)
Intensive care unit	2 (4%)	-1.4 (0.161)	7 (12%)	+1.4 (0.161)
Emergency room	2 (4%)	-*4.5 (<0.001)*	25 (43%)	*+4.5 (<0.001)*

*p*-value (global) < 0.001; Pearson’s chi-square.

Another option for analysis of subgroups is Goodman and Kruskal’s *lambda* test, which is a measure of the proportional reduction in error in the contingency table analysis for multinomial data, indicating the point to which modal categories and frequencies for each value of the independent variable differ from the values of the independent variable.^35^ In the same manner, the table can be partitioned into 2 × 2 subtables. However, the multiple comparisons must be adjusted to control inflation of the type I error, using the Bonferroni procedure, for example.^20^

Epidemiological research often employs dichotomous outcomes (for example, cure, death, sickness) to compare two or more groups (for example, placebo vs. treatment). Characteristics intrinsic to the designs of studies have led to a growing tendency for comparisons of these proportions to be estimated from their epidemiological measures of effect, such as odds ratios, relative risk, or prevalence ratios, rather than merely according to the results of statistical tests of proportion.^36^,^37^ Both the *p*-value and the confidence interval of such associations can be calculated directly for these estimates using logistic, ordinal, multinominal, or Poisson regression models.^38^

The need to adjust results for covariates that are of importance in the causal model (for example, age, sex, smoking) has demanded wider adoption of these regression techniques for analysis of categorical data. Contingencies in the presence of modest samples or rarity of events in one of the categories can be overcome using bootstrapping techniques, resampling data more than 1,000 times. However, since these methods consider the relationships between subcategories, they do not deal adequately with cases in which one category is zero, in contrast with exact statistical techniques (Barnard’s test, for example).

Table 2 shows examples of methods for analysis of comparisons between two hypothetical treatments (surgical vs. conventional) analyzed with tests of comparison of proportions and regression models, according to sample characteristics. In the special case of estimation of the magnitude of the effect of a study (for example, relative risk and odds ratios) in which there were zero occurrences of one of the categorical variables, it is possible to resort to the (artificial) addition of 0.5 units to the outcome of each group.^5^,^39^,^40^

**Table 2:** Hypothetical examples of (two-tailed) comparisons of incidence of death from a disease treated with a surgical procedure or a conventional treatment.

**Examples**	**Statistical test**	**Statistic/effect**	***p*-value**
2 deaths in 100 surgeries (2%)	Pearson’s chi-square	ꭕ^2^ = 11.97; Df = 1	<0.001
vs.			
16 deaths in 100 conventional treatments (16%)	Poisson (robust) regression	RR = 0.13	0.005
		95%CI = 0.03 to 0.53	
1 death in 50 surgeries (2%)	Barnard	Score = 2.45	0.016
vs.			
8 deaths in 50 conventional treatments (16%)	Poisson regression	RR = 0.13	0.046
	(robust; 1000 resampling)	95%CI = 0.01 to 0.43	
Zero deaths in 50 surgeries (0%)	Barnard	Score = 2.95	0.004
vs.			
8 deaths in 50 conventional treatments (186%)		RR = 0.06^a^	0.034
		95%CI = 0.01 to 0.45	

Df = degrees of freedom; RR = relative risk; 95%CI = 95% confidence interval.

a Relative risk calculated after addition of 0.5 units to the outcome in each group: 0.5 deaths among surgeries, 8.5 deaths among conventional treatments.

Comparison of proportions between groups can also be evaluated unidirectionally or bidirectionally (one/two-tailed), since many analyses are by their nature one-directional, such as comparison of mortality rates from a disease among vaccinated and unvaccinated people or tests of non-inferiority between two treatments.^41^ In such cases, the study hypothesis does not contemplate the possibility that the result could be analyzed bidirectionally, since there is only interest in the effect in one direction. One-tailed analyses of proportions do not enjoy consensus among epidemiologists because, although they have greater statistical power and require smaller samples, they increase the likelihood of type I error.^24^ One-tailed analyses are widely used in studies of viability (pilot studies) and in proof-of-concept studies, which are conducted before traditional clinical trials.^42^-^44^

Situations that involve dependent data should be assessed with the McNemar test (2 × 2 tables), Cochran’s Q test (several groups, dichotomous response), or generalized estimating equations. These analyses, in common with use of resampling techniques, one-tailed estimates, regressions and analyses of variables that demand multivariate adjustment, should be supervised by an experienced statistician.

Finally, comparison of categorical variables is a common need in biomedical studies and inferential conclusions can differ depending on the analytical method employed, especially when the frequencies in subgroups are low. The choice of analytical technique requires theoretical grounding and its description must be justified in the methodology in terms of the parameters for its use.
