# Supplementary material for: Comparing categorical variables in clinical and experimental studies
Source: J Vasc Bras. 2022 Apr 1;21:e20210225. doi: 10.1590/1677-5449.20210225 (PMC8992732; doi:10.1590/1677-5449.20210225)
Supplement: Translation

# Translation: Comparação entre variáveis categóricas em estudos clínicos e experimentais

**Authors:** Anna Carolina Miola, Hélio Amante Miot

**Affiliations:** 1 Universidade Estadual Paulista “Júlio de Mesquita Filho” – UNESP, Faculdade de Ciências Médicas e Biológicas de Botucatu, Botucatu, SP, Brasil.

Diversos estudos de natureza quantitativa, tanto em ciências biomédicas quanto sociais, utilizam variáveis qualitativas, também chamadas de categóricas, as quais expressam sua grandeza pela frequência em que cada uma de suas categorias ocorre. Variáveis qualitativas são divididas em dicotômicas (por exemplo, sexo, óbito, cura), ordinais (por exemplo, estadiamento neoplásico, amplitude do pulso, classe funcional, fototipo, risco anestésico) ou politômicas/multinominais (por exemplo, orientação sexual, tipagem ABO, estado civil, religião, raça, tipo de aneurisma, tipo de úlcera crônica)^1^^-^3.

Quando se utilizam variáveis qualitativas, o fenômeno mensurado pode ser representado pelo percentual de ocorrência em cada categoria, e sua comparação entre os subgrupos deve ser realizada de acordo com a proporção que cada classe ocupa na amostra^3^. Há extensa literatura a respeito de técnicas de análise estatística de variáveis qualitativas^4^^-^6; contudo, este texto abordará a comparação de proporções entre variáveis categóricas. A análise comparativa dessas proporções entre subgrupos utiliza conceitos diferentes da chamada estatística paramétrica, apresentando menor poder estatístico (maior erro tipo II) em situações análogas, como quando uma variável quantitativa (por exemplo, idade) é categorizada (por exemplo, < 30 anos, 30–59 anos, ≥ 60 anos)^7^^,^^8^.

Segundo a estatística frequentista^9^, a probabilidade de uma proporção de eventos selecionados aleatoriamente, sem reposição de casos, pode ser generalizada a partir da distribuição qui-quadrado, e o teste qui-quadrado de Pearson baseia-se na diferença entre as frequências encontradas e as idealmente esperadas para cada categoria, podendo ser utilizado para comparar a aderência da amostra a uma distribuição conhecida (por exemplo, comparação com a literatura) ou a independência entre diferentes amostras^10^. Apesar da popularidade do teste qui-quadrado de Pearson, outros métodos como o teste G (razão de verossimilhança) e o teste de Goodman (contrastes de proporções) também são utilizados para comparação de proporções. Todavia, a superioridade absoluta entre eles ainda não foi sistematicamente definida^11^^-^14.

A aderência de uma proporção encontrada pode ser comparada a uma descrição da literatura ou a uma expectativa teórica (por exemplo, expressão de um fenótipo segundo a segregação de um gene)^15^. Exemplificando, Tamega et al.^16^ estudaram os tipos sanguíneos ABO e Rh de 69 pacientes com lúpus eritematoso, comparando-os com a frequência esperada dessas categorias entre os doadores de sangue da instituição. O teste qui-quadrado de Pearson (de aderência) resultou p-valor = 0,081 para as tripagens ABO e p-valor = 0,721 para a tipagem Rh, aceitando a hipótese que tais classes de tipos sanguíneos encontrados não divergiam do esperado, na população local^16^.

Na pesquisa clínico-epidemiológica, é bastante usual que se apresente uma tabela inicial descritiva com os dados demográficos dos subgrupos, a fim de atestar a sua homogeneidade. Por exemplo, Amiri et al.^17^ incluíram 110 casos e 110 controles em um estudo transversal para testar a associação de índices antropométricos e diabetes melito tipo 2. Entre os diabéticos, 75 (51%) eram do sexo feminino, enquanto no grupo controle foram encontradas 72 mulheres (49%). Segundo essa amostra, a diferença de proporção entre os grupos (2%) não foi considerada significativa (p-valor = 0,668) para essa variável dicotômica segundo o teste qui-quadrado de Pearson (de independência).

Apesar de versátil, o teste qui-quadrado de Pearson apresenta performance inadequada (maior erro tipo I) em amostras mais modestas (*n* ≤ 40), especialmente nas situações em que > 20% dos valores esperados for ≤ 5, o que é relativamente frequente no cenário da pesquisa biomédica. Diversos procedimentos são recomendados nessa situação, desde a fusão de categorias para aumentar o valor esperado (por exemplo, dicotomizar as cores de pele como branca vs. não branca, agrupar os tipos sanguíneos menos comuns B com AB), ou mesmo o uso de outros testes estatísticos.

Há intensa discussão acadêmica sobre quais estratégias analíticas devem ser utilizadas para as situações em que o teste qui-quadrado de Pearson for contraindicado; da mesma forma, diferentes testes para dados categóricos podem se comportar diversamente, de acordo com a forma que as variáveis são coletadas (aleatórias ou não), já que grande parte dos estudos não possuem estrutura amostral completamente aleatorizada^18^^-^20. Os testes exatos de Barnard e de Boschloo são exemplos que corrigem essas limitações para tabelas de contingência 2 × 2^21^^,^^22^. Já o teste G (com correção de Williams) pode ser utilizado para comparações multinominais em situações de contraindicação do teste qui-quadrado de Pearson^21^^,^^23^. Estimativas do p-valor (exato) a partir de reamostragens (*bootstrap*) ou simulação de Monte Carlo também são eficientes para sua estimativa em casos de amostras modestas ou subgrupos com baixa expectativa de ocorrência^19^^,^^24^.

O teste exato de Fisher é citado por muitos textos como solução para casos em que o teste qui-quadrado de Pearson não seja indicado, porém, ele inflaciona o erro tipo II, além de se basear em um modelo de probabilidade condicional, diferente do que é usualmente proposto em pesquisa biomédica (totais marginais variáveis)^25^^,^^26^. Da mesma forma, a correção do teste qui-quadrado de Pearson pelo procedimento de Yates, em tabelas 2 × 2, é excessivamente conservadora. O emprego e as interpretações desses testes devem ser parcimoniosos quando resultarem p-valor próximo ao nível de significância^22^^,^^24^.

Em desenhos mais complexos, que envolvam a interação de mais de duas variáveis categóricas ou ajuste multivariado cuja variável dependente seja categórica, outros métodos de análise podem ser utilizados, como a regressão de Poisson (*log-linear*), a regressão logística e a regressão multinominal, que, assim como no teste qui-quadrado de Pearson, são penalizadas pela ocorrência de frequências baixas entre os subgrupos. Por outro lado, métodos multivariados, como a análise de correspondência múltipla, não são afetados pelas contingências dos testes de hipóteses e podem substanciar análises exploratórias para dados categóricos^4^^,^^27^^,^^28^. Em tempo, a problemática ligada à análise de dados ordinais e o cálculo do tamanho amostral para estudos que envolvam proporções já foram abordados anteriormente^2^^,^^29^^-^32.

Quando comparações de variáveis multinominais resultam significativas, cabe saber quais das proporções internas apresentam divergência do esperado, tendo em vista que o resultado do teste (por exemplo, o teste qui-quadrado de Pearson) refere-se ao comportamento global das proporções, devendo-se, então, proceder a análise *post hoc* das subcategorias. A análise de resíduos da tabela de contingência (padronizada e ajustada) é uma estratégia muito empregada, que retorna a estatística *Z* (Zres) para cada proporção encontrada, permitindo a comparação múltipla entre elas ao identificar quais variáveis específicas mais contribuem para o resultado encontrado no teste global^33^. A partir da análise de resíduos da Tabela 1, pode-se concluir que pacientes oncológicos oriundos do ambulatório apresentaram mais diagnósticos tomográficos de tromboembolismo pulmonar incidentais que os originários do pronto-socorro, sem diferenças com as proporções encontradas na enfermaria e UTI^34^.

**Tabela 1:** Análise de resíduos dos dados de Carneiro et al.^34^ quanto à origem dos pacientes oncológicos com tromboembolismo pulmonar (TEP) à tomografia computadorizada de tórax, quando o achado foi incidental ou havia suspeita prévia.

**Origem**	**Sem suspeita de TEP**		**Com suspeita de TEP**	
(*n* = 48)	Zres (p-valor)	(*n* = 60)	Zres (p-valor)
Ambulatorial	28 (59%)	*+5,1 (<0,001)*	7 (12%)	*-5,1 (<0,001)*
Enfermaria	16 (33%)	-0,2 (0,856)	21 (35%)	+0,2 (0,856)
Unidade de Tratamento Intensivo	2 (4%)	-1,4 (0,161)	7 (12%)	+1,4 (0,161)
Pronto-socorro	2 (4%)	-*4,5 (<0,001)*	25 (43%)	*+4,5 (<0,001)*

p-valor (global) < 0,001; qui-quadrado de Pearson.

Outra opção para a análise dos subgrupos é o teste *lambda* de Goodman e Kruskal que se trata de uma medida de redução proporcional no erro na análise de tabela de contingência para dados multinomiais, indicando até que ponto as categorias e frequências modais para cada valor da variável independente diferem dos valores da variável independente^35^. Da mesma forma, a partição da tabela em subtabelas 2 × 2 pode ser realizada. Contudo, as comparações múltiplas devem ser ajustadas para reduzir o inflacionamento do erro tipo I, por exemplo, usando o procedimento de Bonferroni^20^.

A pesquisa epidemiológica utiliza frequentemente desfechos dicotômicos (por exemplo, cura, óbito, adoecimento) para a comparação de dois ou mais grupos (por exemplo, placebo vs. tratamento). Devido à característica intrínseca do desenho dos estudos, há crescente tendência que a comparação dessas proporções seja estimada a partir das suas medidas epidemiológicas de efeito, como razão de chances, risco relativo ou razão de prevalências, e não somente pelos testes estatísticos de proporção^36^^,^^37^. Tanto o p-valor como o intervalo de confiança para essas associações podem ser calculados diretamente para essas estimativas a partir de modelos de regressão logística, ordinal, multinominal ou de Poisson^38^.

A necessidade de ajuste dos resultados por covariáveis de importância no modelo causal (por exemplo, idade, sexo, tabagismo) vem demandando a popularização dessas técnicas de regressão para a análise de dados categóricos, e a contingência diante das amostras modestas ou da raridade de eventos em uma das categorias pode ser transposta por técnicas de *bootstrap*, com mais de 1.000 reamostragens dos dados. Entretanto, como esses métodos ponderam as relações entre as subcategorias, eles não lidam adequadamente quando uma delas é zero, ao contrário das técnicas estatísticas exatas (por exemplo, teste de Barnard).

A Tabela 2 exemplifica formas de análise para comparações de dois tratamentos hipotéticos (cirúrgico vs. convencional) analisados segundo testes de comparação de proporções e modelos de regressão, de acordo com particularidades amostrais. No caso especial, para estimar a dimensão de efeito de um estudo (por exemplo, risco relativo e razão de chances) em que houve zero ocorrências em uma das variáveis categóricas, pode-se recorrer à adição (artificial) de 0,5 unidades nos desfechos de cada grupo^5^^,^^39^^,^^40^.

**Tabela 2:** Exemplos hipotéticos de comparações (bicaudais) da incidência de morte de uma doença tratada com um procedimento cirúrgico ou um tratamento convencional.

**Exemplos**	**Teste estatístico**	**Estatística/efeito**	**p-valor**
2 mortes em 100 cirurgias (2%)	Qui-quadrado de Pearson	ꭕ^2^ = 11,97; GL = 1	<0,001
vs.			
16 mortes em 100 tratamentos convencionais (16%)	Regressão de Poisson (robusta)	RR = 0,13	0,005
		IC 95% = 0,03 a 0,53	
1 mortes em 50 cirurgias (2%)	Barnard	Escore = 2,45	0,016
vs.			
8 mortes em 50 tratamentos convencionais (16%)	Regressão de Poisson	RR = 0,13	0,046
	(robusta; 1000 reamostragens)	IC 95% = 0,01 a 0,43	
Zero mortes em 50 cirurgias (0%)	Barnard	Escore = 2,95	0,004
vs.			
8 mortes em 50 tratamentos convencionais (186%)		RR = 0,06^a^	0,034
		IC 95% = 0,01 a 0,45	

GL = graus de liberdade; RR = risco relativo; IC 95% = intervalo de confiança de 95%.

a Risco relativo calculado após inclusão de 0,5 unidades no desfecho de cada grupo: 0,5 mortes entre as cirurgias, 8,5 mortes entre os tratamentos convencionais.

A comparação de proporções entre grupos também pode ser avaliada de forma uni ou bidirecional (uni/bicaudal), já que muitas avaliações são, por natureza, unidirecionais, como a comparação da taxa de mortalidade em uma doença entre vacinados e não vacinados ou em testes de não inferioridade entre dois tratamentos^41^. Nesses casos, não faz parte da hipótese de pesquisa a possibilidade de que o resultado seja contemplado de forma bidirecional, interessando apenas o efeito em um sentido. Análises unicaudais entre proporções não são consensuais entre os epidemiologistas, porque, apesar de apresentarem maior poder estatístico e demandarem menor amostragem, aumentam a chance de erro tipo I^24^. Análises unicaudais são muito empregadas em estudos de viabilidade (estudos piloto) e em provas de conceito, que ocorrem antes dos ensaios clínicos tradicionais^42^^-^44.

Situações que envolvam dados dependentes devem ser avaliadas pelo teste de McNemar (tabelas 2 × 2), teste Q de Cochran (vários grupos, resposta dicotômica) ou equações de estimativas generalizadas. Tais análises, assim como uso de técnicas de reamostragem, estimativas unicaudais, regressões e análises de variáveis que demandem ajuste multivariado, devem ser supervisionadas por estatístico experiente.

Finalmente, a comparação entre variáveis categóricas é uma demanda frequente em estudos biomédicos e que pode resultar em diferentes conclusões inferenciais de acordo com o método analítico empregado, especialmente quando as frequências nos subgrupos forem baixas. A escolha da técnica de análise exige fundamentação teórica, e sua descrição precisa ser justificada na metodologia, quanto aos parâmetros de uso.
